# Comparison of Intraductal RFA Plus Stent versus Stent-Only Treatment for Unresectable Perihilar Cholangiocarcinoma—A Systematic Review and Meta-Analysis

**DOI:** 10.3390/cancers14092079

**Published:** 2022-04-21

**Authors:** David M. de Jong, Jeska A. Fritzsche, Amber S. Audhoe, Suzanne S. L. Yi, Marco J. Bruno, Rogier P. Voermans, Lydi M. J. W. van Driel

**Affiliations:** 1 Department of Gastroenterology and Hepatology, Erasmus MC Cancer Institute, University Medical Centre Rotterdam, Doctor Molewaterplein 40, 3015 GD Rotterdam, The Netherlands; d.m.dejong@erasmusmc.nl (D.M.d.J.); a.audhoe@erasmusmc.nl (A.S.A.); s.yi@erasmusmc.nl (S.S.L.Y.); m.bruno@erasmusmc.nl (M.J.B.); 2 Department of Gastroenterology and Hepatology, Amsterdam UMC Location University of Amsterdam, Meibergdreef 9, 1105 AZ Amsterdam, The Netherlands; j.a.fritzsche@amsterdamumc.nl (J.A.F.); r.p.voermans@amsterdamumc.nl (R.P.V.); 3 Amsterdam Gastroenterology Endocrinology Metabolism, Meibergdreef 9, 1105 AZ Amsterdam, The Netherlands; 4 Cancer Center Amsterdam, Cancer Treatment and Quality of Life, De Boelelaan 1118, 1081 HV Amsterdam, The Netherlands

**Keywords:** unresectable perihilar cholangiocarcinoma, radiofrequency ablation, endoscopic stent, percutaneous stent, biliary drainage

## Abstract

**Simple Summary:**

In patients with unresectable perihilar cholangiocarcinoma, adequate biliary drainage is essential. Stent patency remains a challenge in these complex patients, as both plastic and metal stent occlusion may occur, necessitating additional drainage procedures. Radiofrequency ablation (RFA) is a promising local treatment that has already proven its usefulness in other malignancies, such as hepatocellular carcinoma. In this meta-analysis and systematic review, we aimed to compare intraductal RFA with stent placement to stent placement alone in patients with unresectable perihilar cholangiocarcinoma. We found that RFA + stent treatment showed a significantly longer overall survival, in comparison to stent-only treatment. Further research is necessary in order to validate these findings to support the implementation of this promising strategy in clinical practice.

**Abstract:**

Background: One of the cornerstones of palliative treatment for unresectable perihilar cholangiocarcinoma is biliary stent placement in order to restore biliary drainage. In this review, the potential added value of RFA with stent placement in comparison to stent placement alone in patients with unresectable perihilar cholangiocarcinoma is analyzed. Methods: We performed a comprehensive online search for relevant articles in November 2021 (PROSPERO ID: CRD42021288180). The primary endpoint was difference in overall survival. Secondary endpoints included overall survival, stent patency and complications. Only studies comparing survival after RFA + stent placement with stent placement alone were included in the meta-analysis. Non-comparative studies or comparative studies describing stent patency only were included in the systematic review. Results: A total of nine studies, including 217 patients with pCCA who underwent RFA + stent placement and 294 patients who underwent stent-only treatment, met the inclusion criteria for the primary endpoint analysis. Direct comparison between the two treatment groups showed a significantly longer overall survival for RFA + stent treatment, with a pooled HR of 0.65 [95% CI, 0.50–0.84, I^2^ = 38%]. When all eligible studies were included, RFA + stent treatment revealed an overall survival of 9.5 months [95% CI, 6.3–12.6], whereas survival for stent-only treatment was 7.0 months [95% CI, 5.7–8.2]. Due to the heterogeneity of the data, no pooled data analysis could be performed on stent patency or complications. Conclusions: RFA + stent placement displays promising potential to prolong survival. However, further research incorporating confounding factors like use of palliative chemotherapy is necessary in order to validate these findings.

## 1. Introduction

Although perihilar cholangiocarcinoma (pCCA) is a relatively rare condition with an incidence of <6 cases per 100,000 people in most countries, its incidence is increasing across the globe [1,2,3]. Currently, surgical resection is the only curative treatment [4,5]. Unfortunately, only about one-fifth of patients qualify for curative resection at presentation [6].

Almost all patients with unresectable pCCA develop bile duct obstruction [7]. The mainstay of palliative treatment is the restoration of biliary drainage, by the endoscopic or percutaneous placement of plastic or (un)covered self-expanding metal stents (SEMSs) [8]. This treatment reduces or relieves jaundice, which not only improves quality of life but is also a prerequisite for the commencement of palliative chemotherapy (pCTx) in most clinical practices. Several studies have compared metal and plastic stents for palliative drainage in unresectable pCCA [9,10,11]. SEMSs are considered superior because of rapid and adequate biliary decompression, fewer re-interventions and a lower adverse event rate [12,13]. However, maintaining stent patency is a challenge as the tumor continues to grow and may cause obstruction of the biliary stent [10,14].

Intraductal radiofrequency ablation (RFA) is considered a promising treatment option to prolong stent patency and possibly survival in patients with malignant biliary obstruction [14,15]. RFA causes local tumor necrosis by the emission of heat generated using a high-frequency alternating current via a bipolar probe. This therapy has already been proven to be beneficial in patients with hepatocellular carcinoma and liver metastases [16,17]. In patients with unresectable intrahepatic CCA, percutaneous trans-hepatic RFA seems to prolong survival time as well [18]. Since the development of flexible catheters, RFA can be performed inside bile ducts by either an endoscopic or percutaneous approach. According to a recent meta-analysis, RFA can significantly improve stent patency and survival in patients with a malignant biliary obstruction [14]. In this study, however, malignant biliary obstruction in patients with distal cholangiocarcinoma, pancreatic head carcinoma and/or gallbladder carcinoma are also included. Studies solely focusing on the effect of intraductal RFA in patients with pCCA are sparse. This is of importance as the local anatomy of the liver hilum and the associated complexity of biliary drainage and survival are different compared to malignant biliary obstruction of the distal common bile duct. Additionally, the risk of obstruction of segmental side branches necessitates the placement of uncovered stents, which differs from distal obstructions in which covered stents are more commonly placed.

We conducted a systematic review and meta-analysis to investigate the overall survival and stent patency of intraductal RFA in combination with a plastic stent or SEMS versus stent placement only for patients with unresectable pCCA.

## 2. Materials and Methods

### 2.1. Selection Criteria and Search Strategy

The reporting of this systematic review follows the recommendations of the PRISMA guidelines [19]. Studies were identified by searching electronic EMBASE, Medline and Cochrane databases, and the last search was performed in November 2021 by two authors (D.J., J.F.). The study was registered in PROSPERO (CRD42021288180). The search terms are listed in the Supplementary files. Studies that evaluated at least either survival or stent patency in patients with unresectable pCCA were included. For the primary endpoint analysis, studies had to evaluate survival in intraductal RFA + plastic stent or SEMS placement in comparison to stent placement only in patients with unresectable pCCA by endoscopic retrograde cholangiopancreatography (ERCP) or by a percutaneous approach (PTC). Eligible studies were randomized clinical trials, case–control studies and comparative cohort studies. Exclusion criteria were reviews and studies containing results from a mixed group of CCA and non-CCA patients from which separate outcome data could not be extracted. For the secondary endpoint analysis, studies reporting median or mean survival and/or the stent patency of RFA+/− stent placement for pCCA patients specifically were included. For secondary outcome measures such as complications, single-arm studies were also included. There were no language, publication date or publication status restrictions.

### 2.2. Outcomes

The primary outcome was difference in overall survival, expressed by hazard ratio. The secondary outcomes were (1) median overall survival, defined as the time from stent placement +/− RFA until death or end of follow-up; (2) stent patency, defined as the interval between the day of initial procedure and the recurrence of symptoms of biliary obstruction and (3) post-procedure complications in pCCA specifically (within 30 days after the procedure).

Eligibility assessment and data extraction were performed independently in a standardized manner by two reviewers (D.J., J.F.). Disagreements between reviewers were resolved in consensus meetings. Authors were contacted for further information if needed.

The following information was extracted systematically from each included study: (1) the characteristics of trial participants (including age, Bismuth–Corlette classification, time from diagnosis to intervention and concomitant pCTx); (2) type of intervention (including type of stent, endoscopic or percutaneous approaches and repeated interventions); (3) outcome measures (including survival time, stent patency and complication rate). The extracted data were cross-referenced between the two reviewers to rule out discrepancies.

### 2.3. Quality Assessment

Two reviewers (D.J., J.F.) independently assessed the quality of the included studies, according to the Newcastle–Ottawa Scale (NOS) quality assessment tool for cohort studies and a modified Jadad score for randomized controlled trials (RCTs) [20,21]. For the single-arm cohort studies or case series, the Joanna Briggs Institute Critical Appraisal Tool was used [22].

### 2.4. Statistical Analysis

For the primary outcome measure, we performed a meta-analysis using the inverse variance method. Heterogeneity was evaluated using the Cochran Q-test and inconsistency index I^2^. Heterogeneity was classified as low (I^2^ = 0–30%), moderate (I^2^ = 30–50%), or substantial (I^2^ > 50%). Hazard rates, ratios and standard errors were calculated based on a normal distribution. Survival and stent patency data were converted from days to months if not reported as such. Statistical analyses were performed using R software version 4.0.1.

## 3. Results

### 3.1. Study Selection

**Figure 1:**
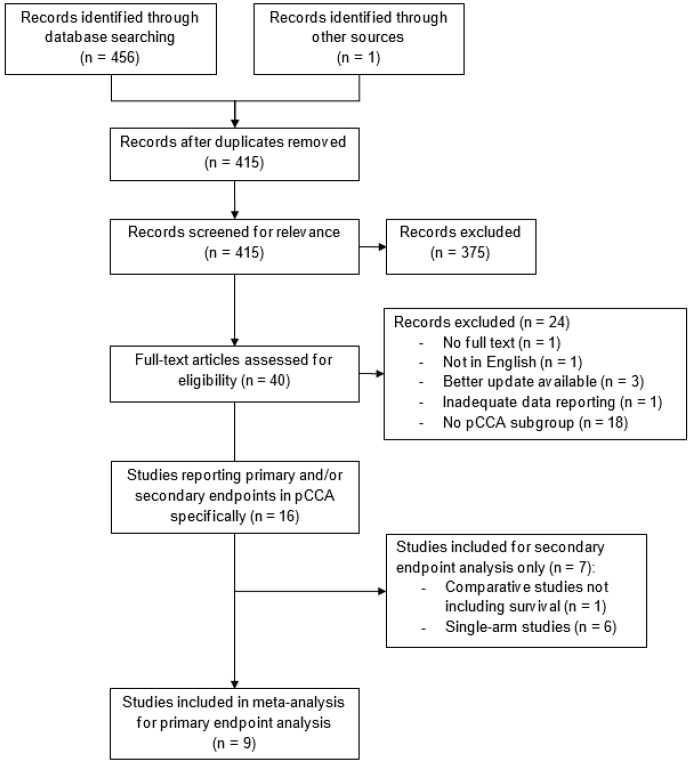
Flowchart of the selection process.

A total of 457 articles were identified. After duplicate removal, 415 articles were screened for relevance. A total of 40 articles remained, of which 24 were excluded for various reasons. Finally, nine articles were included in the meta-analysis for the primary endpoint analysis, as shown in Figure 1. For the secondary endpoint analysis, one additional comparative study reporting stent patency but not survival, and six additional single-arm studies were included.

### 3.2. Baseline Characteristics

In the meta-analysis, a total of 511 patients were included across nine studies [23,24,25,26,27,28,29,30,31]. Of these patients, 217 received RFA + stent placement and 294 underwent stent placement without RFA. Six studies included all four Bismuth–Corlette types of pCCA [24,26,27,29,30,31], as specified in Table 1. Five studies were performed in Asia [24,27,28,29,31], two in the USA [26,30] and two in Europe [23,25].

Five studies used an endoscopic approach [25,26,28,30,31], three used a percutaneous approach only [23,27,29] and one did not specify the method [24]. The type of stents placed were plastic and SEMSs in three studies [25,30,31], SEMSs only in three studies [23,27,29], plastic stents only in two studies [24,28] and one study did not report the type of stent placed [26]. Four studies specified chemo(radio)therapy as additional treatment [23,25,29,30]. The characteristics of the studies included in the meta-analysis are further described in Table 1. The characteristics of the studies included for secondary endpoint analysis only are reported in Supplementary Table S1.

In one study, the protocol included a planned re-intervention. Gao et al. performed a standard re-ERCP with plastic stent replacement +/− re-RFA three months after the initial intervention [28]. The remaining studies did not include a planned re-intervention, but in some studies patients were allowed to undergo re-RFA if indicated—for example, due to in-stent stenosis. The characteristics of the included studies are described further in Table 1.

### 3.3. Quality Assessment

Details regarding quality assessment of the studies included in the meta-analysis are provided in Tables S2 and S3. We found that eight were of good quality [23,25,26,27,28,29,30,31] and one of fair quality [24]. For three studies, only an abstract was published [24,26,30]. Details on quality assessment of the studies included for secondary endpoint analysis only are reported in Tables S3 and S4.

### 3.4. Primary Outcome—Difference in Overall Survival

**Figure 2:**
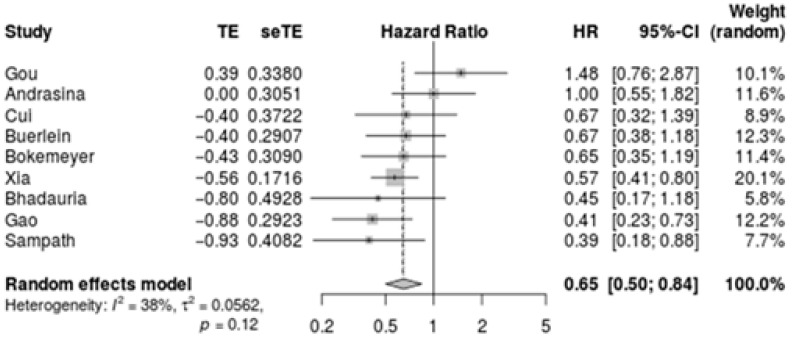
Meta-analysis of the pooled survival hazard ratios. TE = treatment effect, seTE = standard error.

Survival was adequately reported by nine studies [23,24,25,26,27,28,29,30,31]. The overall pooled HR was 0.65 [95% CI, 0.50–0.84, I^2^ = 38%] (Figure 2). These results were consistent after exclusion of the three abstract-only studies [24,26,30]. Bismuth–Corlette types seemed comparable across the two groups: type I (11 vs. 13%), type II (18% vs. 18%), type III (27% vs. 22%) and type IV (44% vs. 48%). However, data were missing for 270 patients across four studies [24,27,29,31]. Plastic stents were used in 47% of patients in the RFA + stent group compared to 50% of patients in the stent-only group, but data were missing or not specified in 238 patients across two studies [26,31].

### 3.5. Secondary Outcomes

#### 3.5.1. Median Survival

When including the eight studies that reported mean or median survival in patients with pCCA, undergoing RFA with stenting showed a median survival of 9.5 months [95% CI, 6.3–12.6], as shown in Figure S1 [23,24,25,26,33,34,35,36]. For the five studies included in the meta-analysis that reported survival data on patients with pCCA in the stent-only group, median survival was 7.0 months [95% CI, 5.7–8.2], as shown in Figure S2 [23,24,25,26,31].

#### 3.5.2. Stent Patency

Stent patency in pCCA specifically was reported by five comparative studies [23,27,29,37,38] and one single-arm study [39]. However, due to the heterogeneous reporting of stent type, placement techniques and the location of biliary obstruction in those studies, a meta-analysis was not possible. Three studies reported on percutaneously placed ucSEMSs in both groups [23,27,29], one study exchanged plastic stents for ucSEMSs by ERCP in both groups [37], one study placed either ucSEMSs or plastic stents by ERCP [39] and one study did not specify what stent was used or how it was placed [38].

Three of the comparative studies reported a significant improvement in stent patency [23,29,38], ranging from a 3.1 to 4.5 month increase. Two of these studies used ucSEMSs [23,27] and in the other study, the type of stent used remained unclear [38]. Two other studies did not find a significant difference [29,37]; one used ucSEMSs [29] and the other study standardly exchanged plastic stents for ucSEMSs [37]. The results of these studies are reported in Table 2.

#### 3.5.3. Complications

Complications were reported specifically for pCCA patients in four comparative studies [26,28,30,38] and three single-arm studies [33,35,36]. As shown in Table 3, none of the comparative studies reported significant differences between the two groups when only pCCA patients were included. The complications most reported on after RFA + stent placement in pCCA patients were cholangitis (0–44%), cholecystitis (10–28%), liver abscesses (10%) and abdominal pain (10–33%). Perforation or pancreatitis were not described after RFA in pCCA patients. All patients with cholecystitis post-procedure were treated with antibiotics or by percutaneous gallbladder drainage. Table S5 shows all reported complications in the included studies, although these were not specifically for pCCA patients only.

## 4. Discussion

In this meta-analysis of 9 studies and systematic review of 16 studies, we compared the efficacy and safety of intraductal RFA + stent to stent-only treatment in patients with unresectable pCCA. The addition of RFA significantly improved survival with a pooled HR of 0.65 [95% CI, 0.50–0.84]. Due to the heterogeneity of the studies, no meta-analysis could be performed for secondary outcome measures, including stent patency and complication rates.

These results are in line with previous reviews regarding intraductal RFA, including all types of malignant biliary obstructions [14,40]. A meta-analysis by Sofi et al., including 505 patients from nine studies, revealed a statistically significant survival advantage for patients treated with RFA as indicated by a pooled HR of 0.72 [95% CI, 0.59–0.87] [14]. Another meta-analysis by Cha et al., including 420 patients from eight studies, came to a similar conclusion with a pooled HR of 0.47 [95% CI, 0.34–0.64] in favor of RFA + stent treatment [40].

The survival of patients with unresectable pCCA is poor, however it varies between studies, with survival ranging from 3 to 10 months [41,42,43]. Therefore, the calculated pooled median survival rates in this systematic review cannot be compared with current literature on overall survival. Furthermore, most patients will not receive RFA early in the disease course, but only when the diagnosis of pCCA has been histologically confirmed and staging has been completed, which may take considerable time. Moreover, the study populations in most studies vary, and therefore it is difficult to compare results considering the potential for confounding factors such as systemic treatment.

There are multiple factors that could influence survival which were inadequately described in the included studies or were not described for pCCA specifically, and hence could not be adjusted for in this meta-analysis. For example, systemic palliative treatment in the form of chemotherapy seems to be of paramount importance since this has been proven to have survival benefit, and in most guidelines the combination of cisplatin/gemcitabine is now presented as the best option for palliative treatment [43,44]. Other palliative treatments, such as radiotherapy or immunotherapy, are increasingly being studied and should therefore also be taken into account [45]. In addition to additional palliative treatment, other factors could also influence survival. A few studies included in this meta-analysis described age ≥ 65 years, number of ERCP procedures and TNM stage IV as poor prognostic factors [26,31]. These have also been reported in previous studies [6,42,43]. Unfortunately, this dataset lacked detailed information on such factors, which made it impossible to further analyze these in detail.

The mechanism of improving survival after RFA is probably explained by improving stent patency due to local tumor ablation. Unfortunately, a pooled analysis was not possible due to the limited amount and heterogeneity of the data, with three of the five studies showing a benefit of RFA regarding stent patency [23,27]. Moreover, there have been preliminary reports on systemic immune mechanisms after RFA that may play a role by modulating circulating immune cells and cytokines. In a mouse model, a weak but detectable immune response was described after RFA. These findings were later confirmed in pancreatic cancer, hepatocellular carcinoma and colorectal liver metastasis [46,47,48]. However, these findings have not yet been confirmed in patients with biliary cancers.

Regarding our other secondary outcome measure (i.e., complications), no pooled analysis could be provided. However, the treatment groups showed no major differences. A limitation present in many reviews on interventions is that the number of complications are routinely reported per patient or per intervention, making direct comparison difficult when patients undergo multiple interventions. Although two studies, including all types of malignant biliary obstruction, found a significantly higher percentage of patients with cholecystitis after RFA + stent placement, this was not reported in any of the other studies [28,31,38]. It is hypothesized that acute cholecystitis can be caused when the cystic duct is included in the RFA tract. This should therefore be avoided whenever possible. Despite the significant difference, the absolute number was very low, and all patients were treated successfully by percutaneous gallbladder drainage and/or antibiotics. Therefore, it can be concluded that RFA + stent placement seems safe, even when crossing of the cystic duct cannot be avoided. In a previous systematic review including all types of malignant biliary strictures, only abdominal pain seemed to occur significantly more often after RFA [14]. In the studies included in our systematic review, abdominal pain was heterogeneously reported and no individual studies reported a significant difference. This is probably partly due to underreporting, considering the large number of retrospective studies that were included.

The main limitation of this systematic review was the inadequate reporting of confounding factors and complications in most of the included studies. Only two RCTs were included, and most studies were of a retrospective design. Although the quality assessment was good for most studies, the specific results of interest were sometimes lacking. Four studies published an abstract only, which included limited information. For example, the type of stent was not reported in two studies. Survival data was reported with significant variation, and manual calculation of the hazard ratios and standard errors was necessary. Furthermore, our findings are limited by a lack of unified treatment strategies in the included studies regarding RFA settings, treatment route and type of stents. Concerning stent patency, and presumably survival as well, the type of stent used is an important factor. Lastly, we excluded studies without a clear description and outcomes for pCCA. However, these studies could have had useful data because some, or even the majority, of the patients were diagnosed with pCCA.

## 5. Conclusions

Despite the limitations and the lack of a clear definite conclusion based on the current literature, this systematic review does indicate the safety and potential benefits of intraductal RFA in patients with unresectable pCCA. Considering the limited palliative treatment options currently available for these patients and the large burden of recurrent jaundice, re-interventions, the concomitant risk of cholangitis and even impaired survival due to recurrent stent obstruction, we believe intraductal RFA could be of great value. Therefore, in order to be able to draw more definite conclusions regarding the benefit of intraductal RFA on survival and stent patency for pCCA patients, RCTs are warranted.

## Figures and Tables

**Table 1:** Characteristics of included articles in meta-analysis for primary endpoint analysis. RFA setting = all included articles performed repeat RFA if segment was too long, R = retrospective, P = prospective, RCT = randomized controlled trial, ERCP = endoscopic retrograde cholangiopancreatography, PTC = percutaneous approach, W = watt, SEMS = self-expanding metal stent, pCTx = palliative chemotherapy, BTx = brachytherapy, RTx = radiotherapy, PDT = photodynamic therapy, HAIC = hepatic arterial infusion chemotherapy. * = Abstract only articles.

Study	Country	Period	Study Design		CCA Type	Intervention	RFA Setting	Stent Type	N (RFA vs. Stent)	Median Survival in Months		Palliative Treatment	
			R/P	Design						RFA	Stent	pCTx	Other
Andrasina [23]	Czech Republic	2010–2019	P	RCT	Bismuth II–IV	PTC	10 W for 90–120 s, Habib	ucSEMS	21 vs. 22	12.3	12.3	14 vs. 13	BTx: 18 vs. 16
Bhadauria [24] *	India	NR	P	Cohort	Bismuth I–IV	NR	8–10 W for 120 s, Habib	Plastic	10 vs. 7	15.8	7.1	NR	NR
Bokemeyer [25]	Germany	2006–2011 controls, 2012–2017 cases	R	Case control	Bismuth III–IV	ERCP	8–10 W for 90 s, Habib	Plastic	17 vs. 20	11.3	7.3	6 vs. 7	NR
								SEMS	3 vs. 2				
Buerlein [26] *	USA	2011–2018	R	Cohort	Bismuth I–IV	ERCP	NR	NR	20 vs. 29	10.0	6.7	NR	PDT: 2 vs. 0
Cui [27,32]	China	2013–2015	R	Cohort	Bismuth I–IV	PTC	10 W for 90 s, Habib	ucSEMS	46 vs. 28	8.0	4.7	NR	NR
Gao [28]	China	2013–2017	P	RCT	Bismuth I–III	ERCP, repeat after 3 months	7–10 W for 90 s, Habib	Plastic	25 vs. 22	HR: 0.414		NR	NR
Gou [29]	China	2013–2018	R	Cohort	Bismuth I–IV	PTC	10 W for 120 s, Habib	ucSEMS	18 vs. 17	HR: 1.480		NR	HAIC: 18 vs 0
Sampath [30] *	USA	2010–2015	R	Cohort	Bismuth I–IV	ERCP	NR	Plastic	8 vs. 10	11.8	4.7	8 vs. 11 (+/−RTx)	NR
								SEMS	2 vs. 5				
Xia [31]	China	2012–2019	R	Matched Cohort	Bismuth I–IV	ERCP	10–12 W for 60–120 s, Habib	Both	47 vs. 132	10.5	6.0	NR	NR

**Table 2:** Stent patency in pCCA patients. NR = not reported, NA = not applicable. * = In 6 of the 20 patients that received a stent (time to occlusion). ^†^ = In 8 of the 22 patients that received a stent (time to occlusion).

Study	Intervention	Stent Type	Group	N	Stent Patency	*p*-Value or HR (95% CI)
Andrasina [23]	PTC	ucSEMS	RFA + stent	20 *	Median 9.6 months [95% CI 5.2–11.2]	0.029
			Stent-only	22 ^†^	Median 4.5 months [95% CI 0.8–10.3]	
Cui [27,32]	PTC	ucSEMS	RFA + stent	25	Median 7.6 months [95% CI 6.8–9.2]	0.009
			Stent-only	14	Median 4.3 months [95% CI 1.7–8.5]	
Gou [29]	PTC	ucSEMS	RFA + stent	18	NR	1.173 [95% CI 0.685–2.011]
			Stent-only	17		
Kang [37]	ERCP	Plastic exchanged for ucSEMS	RFA + stent	13	Median 5.9 months [range 2.0–9.8]	NR
			Stent-only	13	Median 4.0 months [range 3.4–4.6]	
Lee [38]	NR	NR	RFA + stent	21	Median 8.0 months	0.01
			Stent-only	21	Median 4.0 months	
Laleman [39]	ERCP	Both	RFA + stent	9	Median 4.6 months [range: 1.7–11.2]	NA

**Table 3:** Adverse events in pCCA specifically reported <30 days after the procedure in included articles. All adverse events were analyzed per patient. AE = adverse event, NA = not applicable, NR = not reported.

Study		No. per Group	Overall AE Rate	Cholangitis	Cholecystitis	Pancreatitis	Liver Abscess	Bleeding	Abdominal Pain	Perforation	*p*-Value
Buerlein [26]	RFA	20	NR	40%	NR	NR	10%	NR	10%	NR	>0.05
	Stent-only	29	NR	41%	NR	NR	21%	NR	6.9%	NR	
Gao [28]	RFA	25	NR	NR	28%	NR	NR	NR	NR	NR	NR
	Stent-only	22	NR	NR	0%	NR	NR	NR	NR	NR	
Lee [38]	RFA	21	NR	NR	NR	NR	NR	NR	NR	NR	>0.05
	Stent-only	21									
Sampath [30]	RFA	10	NR	30%	NR	NR	NR	NR	NR	0% (bile leak)	NR
	Stent-only	15	NR	0%	NR	NR	NR	NR	NR	7% (bile leak)	
Han [36]	RFA	21	14.3%	0%	10%	0%	NR	0%	NR	0%	NA
Laquière [33]	RFA	12	NR	8%	NR	NR	NR	0%	NR	NR	NA
Wang [35]	RFA	9	NR	44%	NR	0%	NR	0%	33%	0%	NA

## Supplementary Materials

The following supporting information can be downloaded at: https://www.mdpi.com/article/10.3390/cancers14092079/s1, Figure S1: Pooled median survival in months for RFA + stent treatment for studies reporting this in single arm studies specifically for pCCA in combination with articles included in the primary meta-analysis; Figure S2: Pooled median survival in months for stent-only treatment for all studies included in this primary meta-analysis, with stent type and method of stent placement; Search strategy; Table S1: Characteristics and outcome measures of studies describing survival and/or stent patency including the single-arm studies that were included for secondary endpoint analysis only; Table S2: Newcastle–Ottawa Quality assessment scale for cohort studies; Table S3: Modified Jadad scale for RCTs; Table S4: The Joanna Briggs Institute Critical Appraisal Tool for Case Series included in secondary endpoint analysis; Table S5: Adverse events reported in included articles.
