# Supplementary material for: Comparison of Intraductal RFA Plus Stent versus Stent-Only Treatment for Unresectable Perihilar Cholangiocarcinoma—A Systematic Review and Meta-Analysis
Source: Cancers (Basel). 2022 Apr 21;14(9):2079. doi: 10.3390/cancers14092079 (PMC9099890; doi:10.3390/cancers14092079)
Supplement: Supplementary file 1 [file cancers-14-02079-s001.zip › cancers-1673427-supplementary.pdf]

## Search strategy

Embase

('bile duct cancer'/exp OR (klatskin* OR cholangiocarcinom* OR ((bile-duct OR bile-tract OR biliar* OR cholangio* OR gall-duct*) NEAR/3 (carcinom* OR cancer* OR neoplas* OR tumo* OR cholangiocarcinom*))):ab,ti) AND ('radiofrequency ablation'/exp OR (radiofrequency-ablation OR radio-frequency-ablation OR RFA):ab,ti) AND ('stent'/de OR 'biliary stent'/de OR 'metal stent'/exp OR 'nitinol stent'/exp OR 'plastic stent'/de OR 'self expanding stent'/exp OR (stent*):ab,ti)

Medline (Ovid)

(exp Biliary Tract Neoplasms/ OR (klatskin* OR cholangiocarcinom* OR ((bile-duct OR bile-tract OR biliar* OR cholangio* OR gall-duct*) ADJ3 (carcinom* OR cancer* OR neoplas* OR tumo* OR cholangiocarcinom*))).ab,ti.) AND (exp Radiofrequency Ablation/ OR (radiofrequency-ablation OR radio-frequency-ablation OR RFA).ab,ti.) AND (exp Stents/ OR (stent*).ab,ti.)

Web of Science

TS=(((klatskin* OR cholangiocarcinom* OR ((bile-duct* OR bile-tract* OR biliar* OR cholangio* OR gall-duct*) NEAR/2 (carcinom* OR cancer* OR neoplas* OR tumo* OR cholangiocarcinom*)))) AND ( (((radiofrequency OR radio-frequency) NEAR/2 (ablation*)) OR RFA)) AND ((stent*)))

Cochrane

((klatskin* OR cholangiocarcinom* OR ((bile-duct OR bile-tract OR biliar* OR cholangio* OR gall-duct*) NEAR/3 (carcinom* OR cancer* OR neoplas* OR tumo* OR cholangiocarcinom*))):ab,ti) AND ( (radiofrequency-ablation OR radio-frequency-ablation OR RFA):ab,ti) AND ((stent*):ab,ti)

Google Scholar

klatskin|cholangiocarcinoma|'bile|gall duct|tract carcinoma|cancer|neoplasm|tumor|tumour'|'biliary carcinoma|cancer|neoplasm|tumor|tumour' 'radiofrequency|RF ablation'|RFA stent|stents

**Supplementary Table S1.** Characteristics and outcome measures of studies describing survival and/or stent patency including the single arm studies, that were included for secondary endpoint analysis only.

| Study | Country | Period | Study design | | CCA type | Intervention | RFA setting | Stent type | N (RFA) | Palliative treatment | | Outcome | |
|---|---|---|---|---|---|---|---|---|---|---|---|---|---|
| | | | Data collection | Design | | | | | | pCTx | Other | Survival | Stent patency |
| Andrasina [1] | Czech Republic | 2010-2019 | Prospective | RCT | Bismuth II-IV | PTC | 10W for 90-120s, Habib | ucSEMS | 21 | 14 | BTx: 18 | Median 14.7 months (95% CI, 9.6 – 16.6) | Median 9.6 months (range: 5.2 – 12.2) * |
| Bhadauria [2] | India | NR | Prospective | Cohort | Bismuth I-IV | ERCP | 8-10W for 120s, Habib | Plastic | 10 | NR | NR | Median 15.8 months (95% CI, 8.5 – 23.1) | NR |
| Bokemeyer [3] | Germany | 2006-2011 controls, 2012-2017 cases | Retrospective | Case control | Bismuth III-IV | ERCP | 8-10W for 90s, Habib | Both | 20 | 6 | NR | Mean 11.3 months (+/-1.9) | NR |
| Buerlein [4] | USA | 2011-2018 | Retrospective | Cohort | Bismuth I-IV | ERCP | NR | NR | 20 | NR | PDT: 2 | Median 10 months (95% CI, 8.5 – 36.8) | NR |
| Cui [5, 6] | China | 2013-2015 | Retrospective | Cohort | Bismuth I-IV | PTC | 10W for 90s, Habib | ucSEMS | 46 | NR | NR | NR | Median 7.6 months (95% CI 6.8-9.2) |
| Gao [7] | China | 2013-2017 | Prospective | RCT | Bismuth I-III | ERCP, repeat after 3 months | 7-10W for 90s, Habib | Plastic | 25 | NR | NR | HR 0.414 (95% CI, 0.025 – 0.762) | NR |

| | | | | | | | | | | | | | |
|---|---|---|---|---|---|---|---|---|---|---|---|---|---|
| Gou [8] | China | 2013-2018 | Retrospective | Cohort | Bismuth I-IV | PTC | 10W for 120s, Habib | ucSEMS | 18 | NR | HAIC: 18 | HR 1.48 (95% CI, 0.874 – 2.507) | HR 1.173 (95% CI, 0.685 – 2.011) |
| Han [9] | South Korea | NR | Both | Single | Bismuth III-IV | ERCP | 7W for 120s, ELRA Starmed | Both | 21 | 2 | NR | Median 4.9 months (range: 1.7 – 16.1) | NR |
| Kang [10] | South Korea | NR | Prospective | RCT | Bismuth II-IV | ERCP | 7W for 60-120s, ELRA Starmed | Plastic → ucSEMS | 13 | NR | NR | NR | Median 5.9 months (2.0 – 9.8) |
| Laleman [11] | Belgium | 2014-2015 | Prospective | Single | Bismuth III-IV | ERCP | 7-10W for 120s, ELRA Starmed | Both | 9 | NR | NR | NR | Median 4.6 months (range: 1.7 – 11.2) |
| Laquière [12] | France | NR | Retrospective | Single | Bismuth I-IV | ERCP | 10W for 90s, Habib | Both | 12 | 3 | NR | Mean 12.3 months (range: 3 – 31) | NR |
| Lee [13] | South Korea | NR | Prospective | Single | NR | ERCP | ELRA Starmed | NR | 20 | NR | NR | NR | Median 8 months |
| Sampath [14] | USA | 2010-2015 | Retrospective | Cohort | Bismuth I-IV | ERCP | NR | Plastic<br>SEMS | 8<br>2 | 8 (+/- RTx) | NR | Median 11.8 months | NR |
| Tal [15] | Germany | 2012-2013 | Prospective | Single | Bismuth IV | ERCP | 8-10W for 90 second, Habib | Plastic | 7 | NR | NR | Median 6.2 months (IQR: 1.3 – 10.3) | NR |
| Wang [16] | China | 2013-2015 | Prospective | Single | Bismuth III-IV | PTC | 6-10W for 90-120s, Habib | Metal | 9 | 2 | NR | Median 5.3 months (95% CI, 2.5 – 8.1) | NR |

| | | | | | | | | | | | | | |
|---|---|---|---|---|---|---|---|---|---|---|---|---|---|
| Xia [17] | China | 2012-2019 | Retrospective | Matched Cohort | Bismuth I-IV | ERCP | 10-12W for 60-120s, Habib | Both | 47 | NR | NR | Median 10.5 months (95% CI, 8.2 – 12.8) | NR |

***** In 6 out of the 20 patients that received a stent (time to occlusion)

**Supplementary Table S2.** Newcastle-Ottawa Quality assessment scale for cohort studies**.** *Good quality: 7-9, Fair quality: 4-6, Poor quality: 0-3.*

| Article | Bhadauria | Bokemeyer | Buerlein | Cui | Gou | Sampath | Xia |
|---|---|---|---|---|---|---|---|
| **Selection** | | | | | | | |
| 1. Representativeness of the exposed cohort | | | | | | | |
| a. Truly representative | | X | X | X | X | X | X |
| b. Somewhat representative | | | | | | | |
| c. Selected groups | | | | | | | |
| d. No description of derivation of cohort | X | | | | | | |
| 2. Selection of non-exposed cohort | | | | | | | |
| a. Drawn from same community | | X | X | X | X | X | X |
| b. Drawn from different source | | | | | | | |
| c. No description of derivation of non-exposed cohort | X | | | | | | |
| 3. Ascertainment of exposure | | | | | | | |
| a. Secure record | | X | X | X | X | X | X |
| b. Structured interview | | | | | | | |
| c. Written self report | | | | | | | |
| d. No description | X | | | | | | |
| 4. Demonstration that outcome was not present at start of study | | | | | | | |
| a. Yes | X | X | X | X | X | X | X |
| b. No | | | | | | | |
| **Comparability** | | | | | | | |
| 1. Comparability of cohorts on the basis of the design or analysis | | | | | | | |
| a. The study controls for extent of disease | | X | X | X | X | X | X |
| b. Study controls for any additional factor | | X | X | X | X | X | X |
| **Exposure** | | | | | | | |
| 1. Ascertainment of exposure | | | | | | | |
| a. Independent blind assessment | | | | | | | |
| b. Record linkage | X | X | X | X | X | X | X |
| c. Self report | | | | | | | |
| d. No description | | | | | | | |
| 2. Was FU long enough for outcomes to occur? | | | | | | | |
| a. Yes | X | X | X | X | X | X | X |
| b. No | | | | | | | |
| 3. Adequacy of FU of cohort | | | | | | | |
| a. Complete FU | X | X | X | X | | | X |
| b. Subject lost to FU unlikely to introduce bias | | | | | | | |
| c. FU rate <50% and no description | | | | | | X | |
| d. No statement | | | | | X | | |
| **Score** | 4 | 9 | 9 | 9 | 8 | 8 | 9 |

**Supplementary Table S3.** Modified Jadad scale for RCTs. *Good quality: 6-8, Fair quality: 4-5, Poor quality: 0-3.*

| | **Andrasina** | **Gao** | **Kang **** |
|---|---|---|---|
| Was the study described as randomized? | Yes | Yes | Yes |
| Was the method of randomization appropriate? | Yes | Yes | Yes |
| Was the study described as blinded? | No | No* | No |
| Was the method of blinding appropriate? | Not described | Not described | Not described |
| Was there as description of withdrawals and drop-outs? | Yes | Yes | Yes |
| Was there a clear description of the inclusion/exclusion criteria? | Yes | Yes | Yes |
| Was the method used to assess adverse effects described? | Yes | Yes | Yes |
| Was the method of statistical analysis described? | Yes | Yes | Yes |
| **Score:** | **6** | **6** | **6** |

*= Patients and physicians were not blinded, but those responsible for data collection and patients follow-up were.

** = Only included in secondary endpoint analysis

**Supplementary Table S4.** The Joanna Briggs Institute Critical Appraisal Tool for Case Series included in secondary endpoint analysis.

| | **Han** | **Laquière** | **Laleman** | **Lee** | **Tal** | **Wang** |
|---|---|---|---|---|---|---|
| 1. Were there clear criteria for inclusion in the case series? | Yes | Yes | Yes | Yes | Yes | Yes |
| 2. Was the condition measured in a standard, reliable way for all participants included in the case series? | Yes | Yes | Yes | Unknown | Yes | Yes |
| 3. Were valid methods used for identification of the condition for all participants included in the case series? | Yes | Yes | Yes | Unknown | Yes | Yes |
| 4. Did the case series have consecutive inclusion of participants? | No | Yes | Unknown | Unknown | No | No |
| 5. Did the case series have complete inclusion of participants? | NA | NA | NA | NA | NA | NA |
| 6. Was there clear reporting of the demographics of the participants in the study? | Yes | Yes | Yes | No | Yes | Yes |
| 7. Was there clear reporting of clinical information of the participants? | Yes | Yes | Yes | No | Yes | Yes |
| 8. Were the outcomes or follow up results of cases clearly reported? | Yes | Yes | Yes | No | Yes | Yes |
| 9. Was there clear reporting of the presenting site(s)/clinic(s) demographic information? | Yes | Yes | Yes | No | Yes | Yes |
| 10. Was statistical analysis appropriate? | Yes | Yes | Yes | Yes | Yes | Yes |

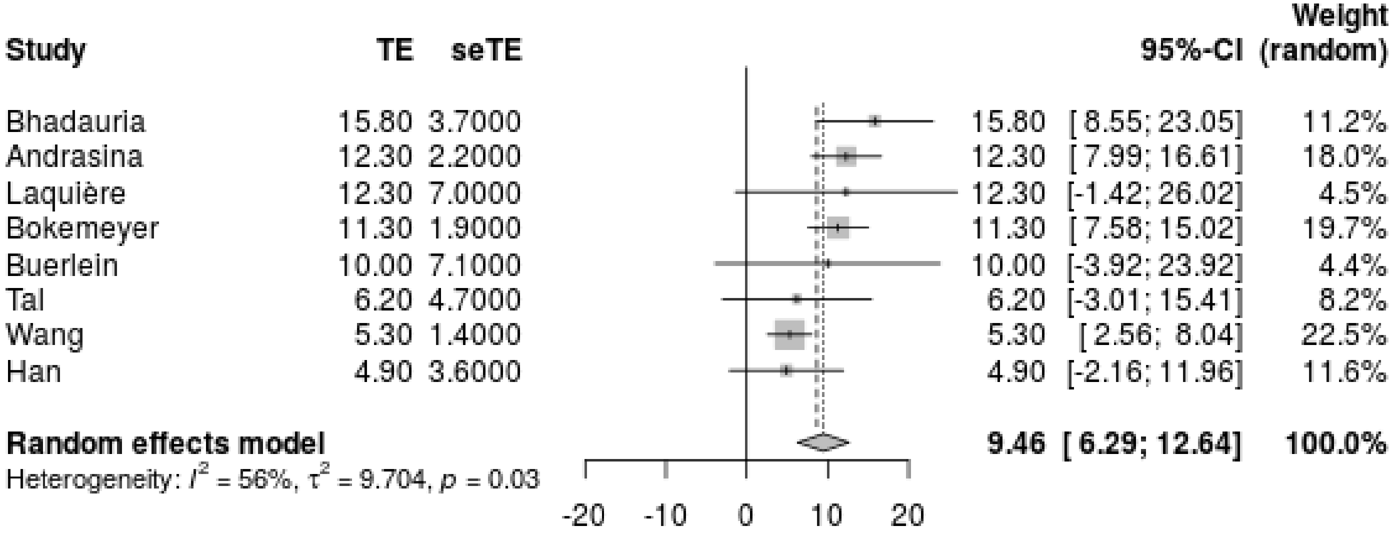


**Supplementary Figure S1.** Pooled median survival in months for RFA+stent treatment for studies reporting this in single arm studies specifically for pCCA in combination with articles included in the primary meta-analysis

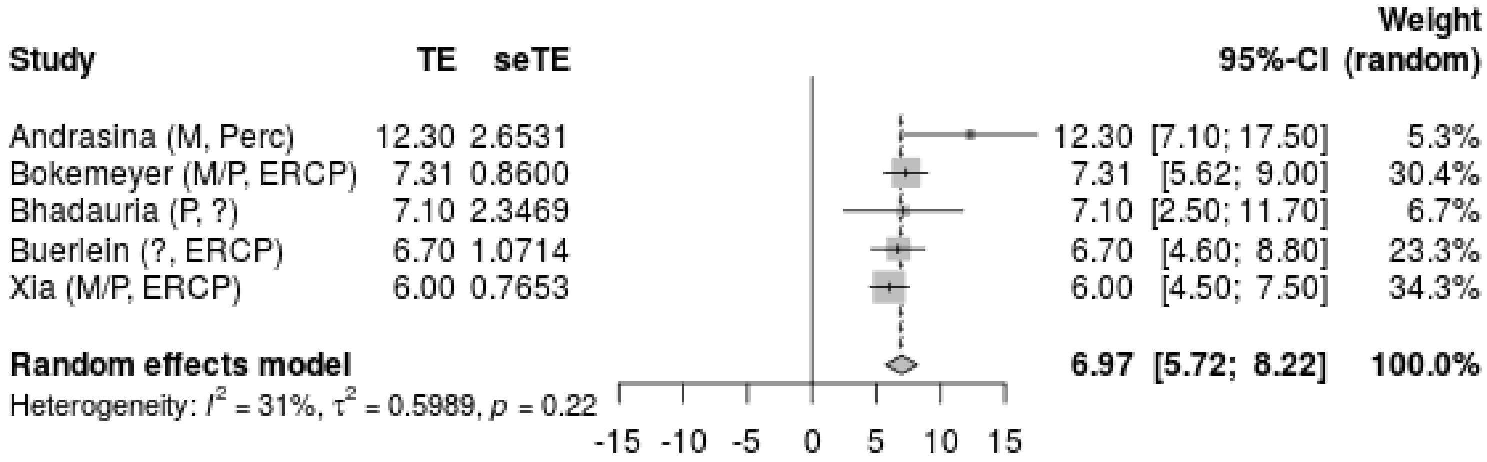


**Supplementary Figure S2.** Pooled median survival in months for stent only treatment for all studies included in this primary meta-analysis, with stent type and method of stent placement

*M = ucSEMS, P = Plastic stents, Perc = Percutaneous stent, ? = Unknown*

**Supplementary Table S5.** Adverse events reported in included articles.

| Study | Analysis | | No. per group | Overall AE rate | Cholangitis | Cholecystitis | Pancreatitis | Liver abscess | Bleeding | Abdominal pain | Perforation | P-value |
|---|---|---|---|---|---|---|---|---|---|---|---|---|
| Andrasina | Per patient | RFA | 21 | NR | NR | NR | NR | NR | NR | NR | NR | 0.062 |
| | | Stent-only | 22 | | | | | | | | | |
| Bhadauria | Per patient | RFA | 17 | NR | 11.8% | NR | NR | NR | 5.9% | More often in RFA | NR | NR |
| | | Stent-only | 15 | NR | 26.7% | NR | NR | NR | 0% | | NR | NR |
| Bokemeyer | Per procedure | RFA | 54 | 18.5% | 11.1% | 3.7% | 1.9% | NR | NR | NR | 1.9% (intestinal) | NR |
| | | Stent-only | NR | NR | NR | NR | NR | NR | NR | NR | NR | |
| Buerlein | Per patient | RFA | 20 | NR | 40% | NR | NR | 10% | NR | 10% | NR | >0.05 |
| | | Stent-only | 29 | NR | 41.4% | NR | NR | 20.7% | NR | 6.9% | NR | |
| Cui | Per procedure | RFA | 50 | NR | 20% | NR | 2% | NR | 14% | 38% | NR | 0.159 |
| | | Stent-only | 39 | NR | 15.4% | 2.6% | 2.6% | NR | 5.2% | 20.5% | NR | |
| Gao | Per patient overall | RFA | 87 | 27.6% | 11.5% | 10.3% | 4.6% | 1.1% | 1.1% | NR | NR | 0.211 |
| | | Stent-only | 87 | 19.5% | 10.3% | 0% | 5.7% | 0% | 3.4% | NR | NR | |
| | Per patient in pCCA specific | RFA | 25 | NR | NR | 28% | NR | NR | NR | NR | NR | NR |
| | | Stent-only | 22 | NR | NR | 0% | NR | NR | NR | NR | NR | |
| Gou | Per patient | RFA | 64 | 67.2% | 12.5% | NR | 10.9% | NR | 14.1% | 21.9% | 1.6% | >0.05 |
| | | Stent-only | 71 | 60.6% | 15.5% | NR | 8.5% | NR | 8.5% | 23.9% | 1.4% | |
| Kang | Per patient | RFA | 15 | NR | 20% | 6.7% | 0% | 0% | 0% | 20% | 0% | >0.05 |
| | | Stent-only | 15 | NR | 33.3% | 6.7% | 6.7% | 6.7% | 0% | 0% | 0% | |

| | | | | | | | | | | | | |
|---|---|---|---|---|---|---|---|---|---|---|---|---|
| Lee | Per patient | RFA | 21 | NR | NR | NR | NR | NR | NR | NR | NR | >0.05 |
| | | Stent-only | 21 | | | | | | | | | |
| Sampath | Per patient | RFA | 10 | NR | 30% | NR | NR | NR | NR | NR | 0% (bile leak) | NR |
| | | Stent-only | 15 | NR | 0% | NR | NR | NR | NR | NR | 6.7% (bile leak) | |
| Xia * | Per patient | RFA | 124 | 19.5% | 6.5% | 4.8% | 8.9% | NR | 1.6% | NR | 0.8% | 0.804 |
| | | Stent-only | 396 | 18.5% | 14.2% | 0.1% | 5.1% | NR | 1.2% | NR | 0% | |

Overall = not specified for cholangiocarcinoma patients, specific = pCCA only.

* = unmatched cohort

## Supplementary References


1. Andrasina, T.; Rohan, T.; Panek, J.; Kovalcikova, P.; Kunovsky, L.; Ostrizkova, L.; Valek, V. The combination of endoluminal radiofrequency ablation and metal stent implantation for the treatment of malignant biliary stenosis—Randomized study. *Eur. J. Radiol.* **2021**, *142*, 109830. https://doi.org/10.1016/j.ejrad.2021.109830.
2. Bhadauria, A.S.; Mohindra, S.; Pandey, G.; Saraswat, V. Early experience with endobiliary radiofrequency ablation (endo-rfa) in patients with unresectable malignant hilar biliary obstruction. *Indian J. Gastroenterol.* **2020**, *34*, 390. https://doi.org/10.1007/s12664-020-01133-9.
3. Bokemeyer, A.; Matern, P.; Bettenworth, D.; Cordes, F.; Nowacki, T.M.; Heinzow, H.; Kabar, I.; Schmidt, H.; Ullerich, H.; Lenze, F. Endoscopic radiofrequency ablation prolongs survival of patients with unresectable hilar cholangiocellular carcinoma—A case-control study. *Sci. Rep.* **2019**, *9*, 13685. https://doi.org/10.1038/s41598-019-50132-0.
4. Buerlein, R.; Strand, D.S.; Patrie, J.T.; Sauer, B.G.; Shami, V.M.; Scheiman, J.M.; Zaydfudim, V.M.; Bauer, T.W.; Adams, R.B.; Wang, A.Y. 544 ercp-directed biliary ablation prolongs survival in patients with unresectable perihilar cholangiocarcinoma compared to stenting alone. *Gastrointest. Endosc*. **2019**, *89*, AB91–AB92. https://doi.org/10.1016/j.gie.2019.04.081.
5. Cui, W.; Wang, Y.; Fan, W.; Lu, M.; Zhang, Y.; Yao, W.; Li, J. Comparison of intraluminal radiofrequency ablation and stents vs. Stents alone in the management of malignant biliary obstruction. *Int. J. Hyperth.* **2017**, *33*, 853–861. https://doi.org/10.1080/02656736.2017.1309580.
6. Cui, W.; Li, J. Iddf2019-abs-0077 percutaneous endobiliary radiofrequency ablation and self-expandable metal stent placement versus stent placement alone for malignant biliary obstruction. *Gut* **2019**, *68*, A129. https://doi.org/10.1136/gutjnl-2019-IDDFAbstracts.255.
7. Gao, D.J.; Yang, J.F.; Ma, S.R.; Wu, J.; Wang, T.T.; Jin, H.B.; Xia, M.X.; Zhang, Y.C.; Shen, H.Z.; Ye, X.; et al. Endoscopic radiofrequency ablation plus plastic stent placement versus stent placement alone for unresectable extrahepatic biliary cancer: A multicenter randomized controlled trial. *Gastrointest. Endosc.* **2021**, *94*, 91–100. https://doi.org/10.1016/j.gie.2020.12.016.
8. Gou, Q.; Wu, L.; Cui, W.; Mo, Z.; Zeng, D.; Gan, L.; He, J.; Mai, Q.; Shi, F.; Chen, M.; et al. Stent placement combined with intraluminal radiofrequency ablation and hepatic arterial infusion chemotherapy for advanced biliary tract cancers with biliary obstruction: A multicentre, retrospective, controlled study. *Eur. Radiol.* **2021**, *31*, 5851–5862. https://doi.org/10.1007/s00330-021-07716-0.
9. Han, S.Y.; Kim, D.U.; Kang, D.H.; Baek, D.H.; Lee, T.H.; Cho, J.H. Usefulness of intraductal rfa in patients with malignant biliary obstruction. *Medicine* **2020**, *99*, e21724. https://doi.org/10.1097/MD.0000000000021724.
10. Kang, H.; Han, S.Y.; Cho, J.H.; Kim, E.J.; Kim, D.U.; Yang, J.K.; Jeon, S.; Park, G.; Lee, T.H. Efficacy and safety of temperature-controlled intraductal radiofrequency ablation in advanced malignant hilar biliary obstruction: A pilot multicenter randomized comparative trial. *J. Hepatobiliary Pancreat. Sci.* **2021**, *00*, 1–10. https://doi.org/10.1002/jhbp.1082.
11. Laleman, W.; van der Merwe, S.; Verbeke, L.; Vanbeckevoort, D.; Aerts, R.; Prenen, H.; van Cutsem, E.; Verslype, C. A new intraductal radiofrequency ablation device for inoperable biliopancreatic tumors complicated by obstructive jaundice: The ignite-1 study. *Endoscopy* **2017**, *49*, 977–982. https://doi.org/10.1055/s-0043-113559.
12. Laquiere, A.; Boustiere, C.; Leblanc, S.; Penaranda, G.; Desilets, E.; Prat, F. Safety and feasibility of endoscopic biliary radiofrequency ablation treatment of extrahepatic cholangiocarcinoma. *Surg. Endosc.* **2016**, *30*, 1242–1248. https://doi.org/10.1007/s00464-015-4322-7.
13. Lee, Y.N.; Moon, J.H.; Park, J.K.; Jo, S.J.; Lee, T.H. Efficacy of endobiliary radiofrequency ablation plus stenting versus stenting alone in patients with inoperable hilar cholangiocarcinoma. *Gut Liver* **2019**, *13*, 19.

14. Sampath, K.; Hyder, S.M.; Gardner, T.; Gordon, S.R. The effect of endoscopic radiofrequency ablation on survival in patients with unresectable perihilar cholangiocarcinoma. *Gastrointest. Endosc.* **2016**, *83*, AB595.
15. Tal, A.O.; Vermehren, J.; Friedrich-Rust, M.; Bojunga, J.; Sarrazin, C.; Zeuzem, S.; Trojan, J.; Albert, J.G. Intraductal endoscopic radiofrequency ablation for the treatment of hilar non-resectable malignant bile duct obstruction. *World J. Gastrointest. Endosc.* **2014**, *6*, 13–19. https://doi.org/10.4253/wjge.v6.i1.13.
16. Wang, Y.; Cui, W.; Fan, W.; Zhang, Y.; Yao, W.; Huang, K.; Li, J. Percutaneous intraductal radiofrequency ablation in the management of unresectable bismuth types iii and iv hilar cholangiocarcinoma. *Oncotarget* **2016**, *7*, 53911–53920. https://doi.org/10.18632/oncotarget.10116.
17. Xia, M.X.; Wang, S.P.; Yuan, J.G.; Gao, D.J.; Ye, X.; Wang, T.T.; Wu, J.; Zhou, D.X.; Hu, B. Effect of endoscopic radiofrequency ablation on the survival of patients with inoperable malignant biliary strictures: A large cohort study. *J. Hepatobiliary Pancreat. Sci.* **2021**, 00, 1–10. https://doi.org/10.1002/jhbp.960.
